# Supplementary material for: Observed feeding behaviours and effects on child weight and length at 12 months of age: Findings from the SPRING cluster-randomized controlled trial in rural India
Source: PLoS One. 2020 Aug 13;15(8):e0237226. doi: 10.1371/journal.pone.0237226 (PMC7425856; doi:10.1371/journal.pone.0237226)
Supplement: S1 Appendix — (DOCX) [file pone.0237226.s001.docx]

**S1 Appendix. Observed Feeding Tool**

| **Identifying information Form type** | | | | |
| --- | --- | --- | --- | --- |
| Cluster | | \|  \|  \|  \| \| --- \| --- \| --- \| | | CLUSTER |
| Village | | \|  \|  \| \| --- \| --- \| | | VILLAGE |
| Household number | | \|  \|  \|  \| \| --- \| --- \| --- \| | | HH |
| Mother name | | | Husband name | |
| Child name | | | Child DOB & Sex (label only) | |
| Woman ID | \|  \|  \|  \| # \|  \|  \| # \|  \|  \|  \| # \|  \|  \|  \|  \| # \|  \|  \| \| --- \| --- \| --- \| --- \| --- \| --- \| --- \| --- \| --- \| --- \| --- \| --- \| --- \| --- \| --- \| --- \| --- \| --- \| | | | WOMANID |
| Child ID | \| CHILD \| # \|  \|  \|  \| # \|  \|  \| # \|  \|  \|  \| # \|  \|  \| # \|  \|  \| \| --- \| --- \| --- \| --- \| --- \| --- \| --- \| --- \| --- \| --- \| --- \| --- \| --- \| --- \| --- \| --- \| --- \| --- \| | | | CHILDID |

| **Visit Information** | | |
| --- | --- | --- |
| Assessor code | \|  \|  \| \| --- \| --- \| | ASSESSOR |
| Date of visit | \| d \| d \| **-** \| M \| M \| M \| **-** \| y \| Y \| y \| y \| \| --- \| --- \| --- \| --- \| --- \| --- \| --- \| --- \| --- \| --- \| --- \| | DATEVISIT |
| Form status | 1. Completed 2. Incomplete (Please specify on ONEYRVISITFORM) 3. Child not yet feeding 4. No meal during day | FORMSTATUS |
| Start Time | \| h \| h \| **:** \| m \| m \| \| --- \| --- \| --- \| --- \| --- \| | TIMESTART |
| End Time | \| h \| h \| **:** \| m \| m \| \| --- \| --- \| --- \| --- \| --- \| | TIMEEND |
| Video Taken | 1. Yes  2. No | VIDEORECORD |
| Video Consent taken | 1. Yes  2. No | VIDEOCONSENT |

Now that you’re feeding [child’s name] I am just going to sit at the side here and focus on my papers. Please ignore me and continue with your feeding as you usually do, and be as normal as possible. I know it’s a bit strange but I’m going to be completely silent because I don’t want to interrupt the feeding. Please don’t worry about me, look at me or talk to me. I also won’t talk to you during the meal.

Take your time with the meal, I’m not in any rush. Whenever you finish just do everything normally as I’ll have some writing to do for 5 minutes after. We’ll talk again after this is finished.

| **A1** | ***Were the child’s hands washed before the meal started?*** | | | 1. Yes 2. No | CHILDWASH |  |
| --- | --- | --- | --- | --- | --- | --- |
| **A2** | ***Did the person feeding wash their hands before the meal started?*** | | | 1. Yes 2. No | MOTHWASH |  |
| **Mouthfuls of food** | | | | | | |
| **B1. Self fed:**  **□ □ □ □ □ □ □ □ □ □ □ □ □ □ □**  **□ □ □ □ □ □ □ □ □ □ □ □ □ □ □** | | \|  \|  \| \| --- \| --- \|   SELFFED | **B2. Mother fed:**  **□ □ □ □ □ □ □ □ □ □ □ □ □ □ □**  **□ □ □ □ □ □ □ □ □ □ □ □ □ □ □** | | \|  \|  \| \| --- \| --- \|   MOTFED | |

| **SELF FEEDING** | | |
| --- | --- | --- |
| **C1. Mother encourages or helps**     \| **✓** \|  \|  \|  \| MOTSELFPOS \| \| --- \| --- \| --- \| --- \| --- \| \| **0** \| **1** \| **2** \| **3+** \| | **C2. Mother discourages or stops**   \| **✓** \|  \|  \|  \| MOTSELFNEG \| \| --- \| --- \| --- \| --- \| --- \| \| **0** \| **1** \| **2** \| **3+** \| | |
| **ENCOURAGEMENT** | | |
| **C3. Mother says things like ‘eat, eat’, ‘chappati is nice’, or ‘you are so good’** *(not in response to child’s request – that would be C5)*   \| **✓** \|  \|  \|  \| MOTVERBALACTPOS \| \| --- \| --- \| --- \| --- \| --- \| \| **0** \| **1** \| **2** \| **3+** \| | | **C4. Mother imitates feeding or plays positive food games**   \| **✓** \|  \|  \|  \| MOTGAMESACTPOS \| \| --- \| --- \| --- \| --- \| --- \| \| **0** \| **1** \| **2** \| **3+** \| |
| **REACTING TO CHILD** | | |
| **C5. Mother responds positively to child’s needs -** for example when child indicates they want food, mother gives food. When child indicates food is too hot, mother makes it cooler.   \| **✓** \|  \|  \|  \| MOTRESPPOSNEEDS \| \| --- \| --- \| --- \| --- \| --- \| \| **0** \| **1** \| **2** \| **3+** \| | **C6. If child seems bored, says ‘no’ or tries to stop feeding: mother tries using a different positive strategy to keep child’s interest**   \| **✓** \|  \|  \|  \| MOTRESPPOSSTRATEGY \| \| --- \| --- \| --- \| --- \| --- \| \| **0** \| **1** \| **2** \| **3+** \| | |
| **HARSHNESS** | | |
| **C7. Mother force feeds, holds child’s head still to give food, shakes child, threatens child, uses an angry tone of voice, shouts or berates child**   \| **✓** \|  \|  \|  \| MOTACTNEG \| \| --- \| --- \| --- \| --- \| --- \| \| **0** \| **1** \| **2** \| **3+** \| | | |

| **CHILD’S INTEREST IN FOOD** | |
| --- | --- |
| **C8. Tries to get food by asking, pointing to food, reaching for food, touching food or opening mouth**   \| **✓** \|  \|  \|  \| CHILDACTPOS \| \| --- \| --- \| --- \| --- \| --- \| \| **0** \| **1** \| **2** \| **3+** \| | **C9. Shows disinterest in having food, e.g says no, sticks out tongue, closes mouth, turns or moves away**   \| **✓** \|  \|  \|  \| CHILDACTNEG \| \| --- \| --- \| --- \| --- \| --- \| \| **0** \| **1** \| **2** \| **3+** \| |

***Instruction to Assessor: Turn over the page as meal is finishing***

|  | ***Were any of the following true when meal ended?*** | |  |  |
| --- | --- | --- | --- | --- |
|  | **D1.1 Child consumed only a few mouthfuls throughout the meal** | | 1. Yes 2. No | FEWMOUTHEND |
|  | **D1.2 Child refused food once and mother ended meal with no additional encouragement** | | 1. Yes 2. No | REFUSED1XEND |
|  | **D1.3 Child refused last two mouthfuls** | | 1. Yes 2. No | REFUSED2XEND |
|  | **D1.4 Meal ended because child was self-feeding and stopped independently** | | 1. Yes 2. No | INDEPENDENTEND |
|  | **D1.5 All Food prepared for child was finished** | | 1. Yes 2. No | FOODFINISHEDEND |
|  | **D1.6 Child looked for more food to eat after meal ended** | | 1. Yes 2. No | STILLHUNGRY |
| **D2** | ***Who was mainly in charge of feeding the child this meal?***  1. Child’s Mother  2. Child’s Grandmother  3. Child’s Father | 4. Child’s Brother  5. Child’s Sister  6. Other Adult  7. Other Child | | FEDCHILD |

**[Ask the next two questions when convenient for the mother]**

| **D3** | Why did you feed [child’s name] at this time today? **[Do not read options]** | 1. Food was ready  2. Other family members eating  3. Child hungry  4. Child always eats at this time  5. Because assessor wanted to see  6. Other – specify ____________  8. Don’t know | WHYFEEDNOW |
| --- | --- | --- | --- |
| **D4** | Do you normally feed [child’s name]? 1. Yes 2. No | | TYPICALFEEDER |
| **D5** | Is this where [child’s name] is normally fed? 1. Yes 2. No | | TYPICALPLACE |
| **D6** | Is this the sort of food that [child’s name] normally eats? 1. Yes 2. No  **[If ‘no’ ask why not and specify below]** | | TYPICALFOOD |
|  | **Specify:** ________________________________________________________________ | | TYPFOODSPECIFY |

Thank you. I will just do a little bit more writing and be finished soon.

***Junior Assessor should manage household environment until Outcome Assessor has completed section E.***

| ***SECTION E*** | | |
| --- | --- | --- |
| ***During the meal, did the mother and child talk about things apart from food, sing songs, touch each other, smile, look at each other, laugh?*** | | |
| **E1** | **Mother**  1. Throughout the meal 2. Sometimes during the meal 3. Not at all | MOTLAUGHTALK |
| **E2** | **Child**  1. Throughout the meal 2. Sometimes during the meal 3. Not at all | CHILDLAUGHTALK |
| **E3** | **Did the mother stop feeding or leave the feeding place during the meal?**  1. Never or one time 2. Two or more times | MOTSTOPLEAVE |
| **E4** | **Did the mother give the child full attention during feeding?**  1. All the time or most of the time 2. Some of the time 3. Not at all | MOTFULLATTN |

| **E5** | ***Did the child have their own plate or bowl?*** | | | | | 1. Yes 2. No | OWNPLATE |
| --- | --- | --- | --- | --- | --- | --- | --- |
| **E6** | ***Approximately how many katoris of food did the child eat? Please circle the amount that they definitely finished.*** | | | | | | KATORIS |
|  | 1. Less than a quarter  2. Quarter  3. Half | 4. Three quarters  5. One  6. One and a quarter | 7. One and a half  8. One and three quarters  9. Two | | | |  |
| **E7** | ***Did any of the following people eat with the child?*** | | | | | | EATWMOTHER  EATWGMOTHER  EATWFATHER  EATWBROTHER  EATWSISTER  EATWOTHER |
|  | Child’s Mother  Child’s Grandmother  Child’s Father  Child’s Brother  Child’s Sister  Other Family Member | | | | 1. Yes 2. No  1. Yes 2. No  1. Yes 2. No  1. Yes 2. No  1. Yes 2. No  1. Yes 2. No | |  |
| **E8** | ***Was feeding done in one place or many places?***  1. One place  2. Many places – mother following child around  3. Many places – mother moving child from place to place | | | | | | FEEDPLACES |
| **E9** | ***Record all the places in which the meal took place:*** | | | | | | |
|  | E9.1 Inside a room in the house | | | 1. Yes 2. No | | | FEEDHOUSE |
|  | E9.2 Inside the courtyard or on the verandah (paved floor) | | | 1. Yes 2. No | | | FEEDPAVED |
|  | E9.3 Inside the courtyard or on the verandah (mud or dust floor) | | | 1. Yes 2. No | | | FEEDMUD |

| ***E10*** | ***Which of the following food items were offered to the child?*** | | |
| --- | --- | --- | --- |
| E10.1 | Roti / chappati | 1. Yes 2. No | OBSCHAPPATI |
| E10.2 | Rice | 1. Yes 2. No | OBSRICE |
| E10.3 | Sabzi – first type | 1. Yes 2. No | OBSSABZI1 |
| E10.4 | Sabzi – second type | 1. Yes 2. No | OBSSABZI2 |
| E10.5 | Daal | 1. Yes 2. No | OBSDAAL |
| E10.6 | Cudhi | 1. Yes 2. No | OBSCUDHI |
| E10.7 | Dalia | 1. Yes 2. No | OBSDALIA |
| E10.8 | Khichdi | 1. Yes 2. No | OBSKICHIDI |
| E10.9 | Uncooked vegetables | 1. Yes 2. No | OBSVEGRAW |
| E10.10 | Uncooked fruit | 1. Yes 2. No | OBSFRUITRAW |
| E10.11 | Yoghurt | 1. Yes 2. No | OBSYOGHURT |
| E10.12 | Eggs | 1. Yes 2. No | OBSEGGS |
| E10.13 | Meat | 1. Yes 2. No | OBSMEAT |
| E10.14 | Fish, prawns or seafood | 1. Yes 2. No | OBSFISH |
| E10.15 | Other – specify: __________________________ | | OBSOTHERFOOD1 |
| E10.16 | Other – specify: __________________________ | | OBSOTHERFOOD2 |
| E10.17 | Other – specify: __________________________ | | OBSOTHERFOOD3 |
