# Supplementary material for: Observed feeding behaviours and effects on child weight and length at 12 months of age: Findings from the SPRING cluster-randomized controlled trial in rural India
Source: PLoS One. 2020 Aug 13;15(8):e0237226. doi: 10.1371/journal.pone.0237226 (PMC7425856; doi:10.1371/journal.pone.0237226)
Supplement: S2 Appendix — (DOCX) [file pone.0237226.s002.docx]

**S2 Appendix. Observed Feeding – Standard Operating Procedures**

**Overview**

You will use this tool/form to record what happens during a child’s mealtime on the day you are in the household. It is a combination of questions for you to answer, observations for you to tick when you see them and questions for you to ask the mother. You should work through the pages in the order they are written. This means you will finish one page before moving to the next.

**Important information about starting the observation**

You will be in the household for much of the day so should have an opportunity to see most children eating one meal. Arrange a time with the mother and caregivers that they think is convenient and make sure you are there at that time.

Find a place where you can sit out of the way, so you are not distracting the person doing the feeding or the child. The ideal position is one where you can see the child and feeder’s faces but they are not looking directly at you. The JOA1 will help to avoid people distracting you but should also try and stay out of the way as much as possible so that the feeder and child feel as relaxed and normal as possible.

Say to mother,

“*Now that you’re feeding [child’s name] I am just going to sit at the side here and focus on my papers. Please ignore me and continue with your feeding as you usually do, and be as normal as possible. I know it’s a bit strange but I’m going to be completely silent because I don’t want to interrupt the feeding. Please don’t worry about me, look at me or talk to me. I also won’t talk to you during the meal.*

*Take your time with the meal, I’m not in any rush. Whenever you finish just do everything normally as I’ll have some writing to do for 5 minutes after. We’ll talk again after this is finished.”*

**Page 1**

| **A1** | ***Were the child’s hands washed before the meal started?*** | This washing must be done with water and must have been done in the few minutes prior to the feeding starting to count as a ‘yes’ |
| --- | --- | --- |
| **A2** | ***Did the person feeding wash their hands before the meal started?*** |  |

You should complete ‘Start Time’ in the visit information table when the mother and child are settled in the place in which they are going to feed and the first mouthful of food is offered to the child.

Then you should turn over to page 2.

**Page 2**

You should keep page 2 open from the moment the feeding session starts till the moment it ends. You should not go on to page 3 until you are happy that the feeding session has ended and you have written the ‘End Time’ in the visit information table.

Page 2 is a list of actions that you need to observe. You should tick each time you see the action, as follows:

| **Mouthfuls of food** | |
| --- | --- |
| **B1. Self fed:**  **□ □ □ □ □ □ □ □ □ □ □ □ □ □ □**  **□ □ □ □ □ □ □ □ □ □ □ □ □ □ □** | For this item you need to watch carefully and mark a ✓ into a **□** every time you see a child (B1) or mother (B2) inserting a mouthful into the child’s mouth. It doesn’t matter if this is a small or a big mouthful. It also doesn’t matter if this falls or is spit out. They all count as a 1 ✓. Keep doing this until the meal is finished.  Note: You should always tick the first box on the first row first and then tick each box on that row in order before moving onto the next row. |
| **B2. Mother fed:**  **□ □ □ □ □ □ □ □ □ □ □ □ □ □ □**  **□ □ □ □ □ □ □ □ □ □ □ □ □ □ □** |  |
| \|  \|  \| \| --- \| --- \|   SELFFED | Right at the end of the session you will come back to add up your ✓ marks and insert a number in each of these boxes.  For example, if you ticked 12 times in self fed, you would enter 12. If you ticked 5 times you would enter 05. |
| \|  \|  \| \| --- \| --- \|   MOTFED |  |

For items C1 – C9 you should tick the box each time you see this action happening, to a maximum of 3 times. After three times you can stop ticking that item and focus on observing the others.

| **SELF FEEDING** | |
| --- | --- |
| **C1. Mother encourages or helps**     \| **✓** \|  \|  \|  \| MOTSELFPOS \| \| --- \| --- \| --- \| --- \| --- \| \| **0** \| **1** \| **2** \| **3+** \| | Self-feeding is where the child puts food into their own mouth. The mother can encourage and help the child to do this (C1) for example by giving food to the child to eat themselves or by saying “well done” or clapping.  The mother can also discourage or stop the child (C2), for example by saying ‘no’, taking food away from the child when they try to pick it up or by clearly stopping the child from picking up food which they want to eat. Stopping a child from pointing at food does not count. |
| **C2. Mother discourages or stops**   \| **✓** \|  \|  \|  \| MOTSELFNEG \| \| --- \| --- \| --- \| --- \| --- \| \| **0** \| **1** \| **2** \| **3+** \| |  |

| **ENCOURAGEMENT** | |
| --- | --- |
| **C3. Mother says things like ‘eat, eat’, ‘chappati is nice’, or ‘you are so good’** *(not in response to child’s request – that would be C5)*   \| **✓** \|  \|  \|  \| MOTVERBALACTPOS \| \| --- \| --- \| --- \| --- \| --- \| \| **0** \| **1** \| **2** \| **3+** \| | Tick if the mother says encouraging things like this to try to help the child to eat. These are all things that come from the mother – so anything that is in response to a child’s request will come under C5.  Note also that encouragement for self-feeding should only be coded in C1 and not here in C3. |
| **C4. Mother imitates feeding or plays positive food games**   \| **✓** \|  \|  \|  \| MOTGAMESACTPOS \| \| --- \| --- \| --- \| --- \| --- \| \| **0** \| **1** \| **2** \| **3+** \| | Tick if the mother does either of these things to try to encourage the child to eat.  Imitating means the mother pretends to feed, and shows the child how to eat.  Food games include moving food around as part of a game, telling positive stories about food items and hiding and uncovering food for example. |

| **REACTING TO CHILD** | |
| --- | --- |
| **C5. Mother responds positively to child’s needs -** for example when child indicates they want food, mother gives food. When child indicates food is too hot, mother makes it cooler.   \| **✓** \|  \|  \|  \| MOTRESPPOSNEEDS \| \| --- \| --- \| --- \| --- \| --- \| \| **0** \| **1** \| **2** \| **3+** \| | Tick if you see the mother respond to the child’s needs. Apart from the examples given, the child might also indicate that they would like a drink of water, that they are uncomfortable or that they would like someone else to feed them. If any of these things happen and the mother reacts in a way which helps feeding then tick here.  All ticks here mean that the mother’s response to the child helped feeding. If, for example, the child indicates that they want to go outside and the mother takes the child and this interrupts feeding then do not tick here. |
| **C6. If child seems bored, says ‘no’ or tries to stop feeding: mother tries using a different positive strategy to keep child’s interest**   \| **✓** \|  \|  \|  \| MOTRESPPOSSTRATEGY \| \| --- \| --- \| --- \| --- \| --- \| \| **0** \| **1** \| **2** \| **3+** \| | Only tick in this item if the child first seems bored, says ‘no’ or tries to stop feeding and the mother tries a different strategy to keep them interested.  These strategies might include: giving the child new instructions, focussing the child’s attention onto something new, asking them a question, diverting them briefly, showing them how to eat, changing the food being offered or offering praise. |

| **HARSHNESS** | |
| --- | --- |
| **C7. Mother force feeds, holds child’s head still to give food, shakes child, threatens child, uses an angry tone of voice, shouts or berates child**   \| **✓** \|  \|  \|  \| MOTACTNEG \| \| --- \| --- \| --- \| --- \| --- \| \| **0** \| **1** \| **2** \| **3+** \| | Mark here if any of these things happen. Force-feeding involves the mother forcing food into a child’s mouth against their will and when they are refusing to accept it.  Any shaking or threatening counts whether a large or small action.  Any use of an angry tone of voice, shouting or berating counts. |

| **CHILD’S INTEREST IN FOOD** | |
| --- | --- |
| **C8. Tries to get food by asking, pointing to food, reaching for food, touching food or opening mouth**   \| **✓** \|  \|  \|  \| CHILDACTPOS \| \| --- \| --- \| --- \| --- \| --- \| \| **0** \| **1** \| **2** \| **3+** \| | Tick here if you observe the child doing any of these things. |
| **C9. Shows disinterest in having food, e.g says no, sticks out tongue, closes mouth, turns or moves away**   \| **✓** \|  \|  \|  \| CHILDACTNEG \| \| --- \| --- \| --- \| --- \| --- \| \| **0** \| **1** \| **2** \| **3+** \| |  |

You will sometimes see two actions that fall under one category happening soon after one another, in these cases you should tick twice. Occasionally, one action might last a long time – most likely in C2,4,7 and 9 – in these cases you should tick each time an action lasts for around 15 seconds. This means if it lasts for 16-30 seconds you should tick twice and for 31 seconds or more tick the 3+ box.

**After the meal has finished you should go to page 1 and fill in the ‘End Time’. Then go to page 3. After turning to page 3 you should not return to this page.**

**Page 3**

**The following questions should not be read out. You should answer them immediately after turning onto this page.**

| **D1** | | ***Were any of the following true when feeding ended?***  Answer ‘yes’ or ‘no’ to each of these questions.  The meal ending is defined as the mother stopping feeding. Usually this will be accompanied by the mother getting up from where she is sitting, or placing the plate or bowl of food to one side.  When you think the meal is finished, wait for approximately 30 seconds to make sure that it doesn’t restart before answering the following questions. | | | |
| --- | --- | --- | --- | --- | --- |
|  | | **D1.1 Child consumed only a few mouthfuls throughout the meal** | | | This means that the child ate 4 or fewer mouthfuls of food when the meal ended |
|  |  | **D1.2 Child refused food once and mother ended meal with no additional encouragement** | | | This encouragement can be either verbal or through an action including attempting to feed child |
|  |  | **D1.3 Child refused last two mouthfuls** | | | If the child refused the last two mouthfuls or more this should be yes. |
|  |  | **D1.4 Meal ended because child was self-feeding and stopped independently** | | |  |
|  |  | **D1.5 All Food prepared for child was finished** | | |  |
|  |  | **D1.6 Child looked for more food to eat after meal ended** | | | This means that after the meal was finished and, for example, the mother got up or the food was put away, the child went in search of more food, pointed to food or asked for more food. |
| **D2** | | ***Who was mainly in charge of feeding the child this meal?***  1. Child’s Mother  2. Child’s Grandmother  3. Child’s Father | | 4. Child’s Brother  5. Child’s Sister  6. Other Adult  7. Other Child | Select the one person who did most of the feeding |

**Next you should ask D3 to D6 to the person you selected in above in D2.**

| **D3** | How did you decide to feed [child’s name] at this time today? **[Do not read options]** | 1. Food was ready  2. Other family members eating  3. Child hungry  4. Child always eats at this time  5. Because assessor wanted to see  6. Don’t know  7. Other – specify:_____________ | Do not read the options, select the most appropriate answer |
| --- | --- | --- | --- |
| **D4** | Do you normally feed your child? 1. Yes 2. No | | If the answer is that different people feed the child each day, select ‘yes’ if this sometimes include the person who fed today. That is, it is not unusual for them to feed the child. |
| **D5** | Is this where [child’s name] is normally fed? 1. Yes 2. No | |  |
| **D6** | Is this the sort of food that [child’s name] normally eats?  1. Yes 2. No **[If ‘no’ ask why not and specify below]** | | ‘Sort of food’ means that the child normally eats similar meals to the one you have just seen. It doesn’t have to be the exact items.  For example, if the child eats roti-sabzi or dalia every day but today ate non-vegetarian then the answer would be ‘no’ and this would be explained in ‘specify’. |
|  | **Specify:** _____________________________________________ | |  |

Now say “Thank you. I will just do a little bit more writing and be finished soon” and turn to complete the questions on page 4 and 5 which are for you to answer.

| ***SECTION E*** | | |
| --- | --- | --- |
| ***During the meal, did the mother and child talk about things apart from food, sing songs, touch each other, smile, look at each other, laugh?*** | | Answer depending on whether you saw these things happening throughout the meal (1), sometimes (2) or not at all (3). |
| **E1** | **Mother**  1. Throughout the meal 2. Sometimes during the meal 3. Not at all |  |
| **E2** | **Child**  1. Throughout the meal 2. Sometimes during the meal 3. Not at all |  |
| **E3** | **Did the mother stop feeding or leave the feeding place during the meal?**  1. Never or one time 2. Two or more times | Answer depending on whether you saw these things happening never or one time (1) or 2 or more times (2). |
| **E4** | **Did the mother give the child full attention during feeding?**  1. All the time or most of the time 2. Some of the time 3. Not at all | Answer depending on whether you think the mother gave the child her full attention during feeding – all the time or almost all the time (1), most of the time (2) or not at all (3) |

| **E5** | ***Did the child have their own plate or bowl?*** | | | 1. Yes 2. No | | ‘Yes’ here means the child’s plate or bowl was not shared with any other person eating at the same time.  If they are given food from elsewhere this is ok but the important thing is that the child’s plate or bowl is not shared with others. |
| --- | --- | --- | --- | --- | --- | --- |
| **E6** | ***Approximately how many katoris of food did the child eat? Please circle the amount that they definitely finished.*** | | | | | Use your training and select the amount of food the child finished compared to the standard size katori which you have been issued. |
|  | 1. Less than a quarter  2. Quarter  3. Half | 4. Three quarters  5. One  6. One and a quarter | | | 7. One and a half  8. One and three quarters  9. Two |  |
| **E7** | ***Did any of the following people eat with the child?*** | | | | | Answer each person with a ‘yes’ or ‘no’.  These people must have themselves eaten food to count. Do not select ‘yes’ if the person was present but did not eat. |
|  | Child’s Mother  Child’s Grandmother  Child’s Father  Child’s Brother  Child’s Sister  Other Family Member | | | 1. Yes 2. No  1. Yes 2. No  1. Yes 2. No  1. Yes 2. No  1. Yes 2. No  1. Yes 2. No | |  |
| **E8** | ***Was feeding done in one place or many places?***  1. One place  2. Many places – mother following child around  3. Many places – mother moving child from place to place | | | | | Choose one option. If the child was moving around but the food was always eaten in one place select ‘1’, ‘One place’. |
| **E9** | ***Record all the places in which the meal took place:*** | | | | | |
|  | E3.1 Inside a room in the house | | 1. Yes 2. No | | | Answer each question with a ‘yes’ or ‘no’. 1,2 or 3 options can be ‘1’, ‘Yes’ for this question. |
|  | E3.2 Inside the courtyard or on the verandah (paved floor) | | 1. Yes 2. No | | |  |
|  | E3.3 Inside the courtyard or on the verandah (mud or dust floor) | | 1. Yes 2. No | | |  |

| ***E10*** | ***Which of the following food items were offered to the child?*** | | Answer each item with a ‘yes’ or ‘no’ depending on whether it was offered to the child to eat. It doesn’t matter if the child actually ate the food item or not |
| --- | --- | --- | --- |
| E10.1 | Roti / chappati | 1. Yes 2. No |  |
| E10.2 | Rice | 1. Yes 2. No | If rice was only eaten as a part of a ‘kichidi’ dish then mark ‘no’ here and select ‘yes’ for ‘kichidi’ in E10.8. |
| E10.3 | Sabzi – first type | 1. Yes 2. No | Select ‘1’, ‘yes’ if the child was offered one sabzi. |
| E10.4 | Sabzi – second type | 1. Yes 2. No | Select ‘1’, ‘yes’ only if the child was offered a second sabzi (ie 2 in total) |
| E10.5 | Daal | 1. Yes 2. No |  |
| E10.6 | Cudhi | 1. Yes 2. No |  |
| E10.7 | Dalia | 1. Yes 2. No |  |
| E10.8 | Kichidi | 1. Yes 2. No |  |
| E10.9 | Uncooked vegetables | 1. Yes 2. No |  |
| E10.10 | Uncooked fruit | 1. Yes 2. No |  |
| E10.11 | Yoghurt | 1. Yes 2. No |  |
| E10.12 | Eggs | 1. Yes 2. No |  |
| E10.13 | Meat | 1. Yes 2. No |  |
| E10.14 | Fish, prawns or seafood | 1. Yes 2. No |  |
| E10.15 | Other – specify: __________________________ | | Use this box if the child was offered any other foods, write as many as they were offered. Do not include drinks. |
| E10.16 | Other – specify: __________________________ | |  |
| E10.17 | Other – specify: __________________________ | |  |
